# TB DEPOT (Data Exploration Portal): A multi-domain tuberculosis data analysis resource

**DOI:** 10.1371/journal.pone.0217410

**Published:** 2019-05-23

**Authors:** Andrei Gabrielian, Eric Engle, Michael Harris, Kurt Wollenberg, Octavio Juarez-Espinosa, Alexander Glogowski, Alyssa Long, Lisa Patti, Darrell E. Hurt, Alex Rosenthal, Mike Tartakovsky

**Affiliations:** Office of Cyber Infrastructure & Computational Biology, National Institute of Allergy and Infectious Disease, National Institutes of Health, Bethesda, MD, United States of America; Tianjin University, CHINA

## Abstract

The NIAID TB Portals Program (TBPP) established a unique and growing database repository of socioeconomic, geographic, clinical, laboratory, radiological, and genomic data from patient cases of drug-resistant tuberculosis (DR-TB). Currently, there are 2,428 total cases from nine country sites (Azerbaijan, Belarus, Moldova, Georgia, Romania, China, India, Kazakhstan, and South Africa), 1,611 (66%) of which are multidrug- or extensively-drug resistant and 1,185 (49%), 863 (36%), and 952 (39%) of which contain X-ray, computed tomography (CT) scan, and genomic data, respectively. We introduce the Data Exploration Portal (TB DEPOT, https://depot.tbportals.niaid.nih.gov) to visualize and analyze these multi-domain data. The TB DEPOT leverages the TBPP integration of clinical, socioeconomic, genomic, and imaging data into standardized formats and enables user-driven, repeatable, and reproducible analyses. It furthers the TBPP goals to provide a web-enabled analytics platform to countries with a high burden of multidrug-resistant TB (MDR-TB) but limited IT resources and inaccessible data, and enables the reusability of data, in conformity with the NIH’s Findable, Accessible, Interoperable, and Reusable (FAIR) principles. TB DEPOT provides access to “analysis-ready” data and the ability to generate and test complex clinically-oriented hypotheses instantaneously with minimal statistical background and data processing skills. TB DEPOT is also promising for enhancing medical training and furnishing well annotated, hard to find, MDR-TB patient cases. TB DEPOT, as part of TBPP, further fosters collaborative research efforts to better understand drug-resistant tuberculosis and aid in the development of novel diagnostics and personalized treatment regimens.

## Introduction

Tuberculosis (TB) is a major challenge for scientists, clinicians, and public health professionals alike. An estimated one-third of the world's population is carrying latent tuberculosis (TB) [1], and as one of the top ten causes of death worldwide, TB was responsible for 1.6 million deaths in 2017 [2]. Although the disease is curable, treatment involves adherence to six months of a multi-drug regimen.

The emergence of drug-resistant (DR) and multidrug-resistant (MDR) forms of TB add additional difficulties for addressing TB globally. Its treatment is toxic and lengthy (at least five drugs must be taken for a duration of 9–24 months [3], and both the outcomes and relapse rates [2] are poor. Globally, only 55% of MDR-TB cases that initiated treatment had successful outcomes. Treatment success is even lower for extensively drug-resistant TB (XDR-TB) cases—those not responding to most second-line treatment drugs—for which treatment success was 34% [2]. In 2009 Velayati et al introduced a new term, Totally Drug-Resistant Tuberculosis (TDR-TB), reporting several instances of patients that were resistant to all first- and second-line drugs tested [4]. There is a distinct possibility of drug-resistant strains of *Mycobacterium tuberculosis* (*M*.*tb*) eventually becoming dominant, replacing the drug-sensitive strains and starting a new wave of TB epidemics worldwide [5].

Due to the changing nature of the TB threat and its continually evolving pathogen, it is critical that TB diagnostics and treatment be improved, especially for MDR-TB. Doing so requires a comprehensive understanding of the complexity of the TB disease, including the wide array of biological, social, and clinical factors that influence its activation, transmission, infection rate, and treatment outcomes. Important insights can be obtained from the analysis of existing data associated with the treatment and care of TB patients. Diverse information that is routinely collected from TB patients to diagnose and treat the disease appropriately, including lab tests, microbiology tests, culture tests, frontal chest X-ray (CXR) and/or computed tomography (CT) scans, can be augmented with *M*.*tb* genomic information, patient microbiome data, and host genomic markers related to drug metabolism and immune system condition. Analysis of the above-mentioned data in the context of treatment history and outcomes can be used to optimize drug regimens and diagnostics, both at the beginning and end of treatment. However, collecting enough sufficiently-detailed TB patient cases for statistical analysis is difficult, especially from rare DR-TB cases.

During 2012, the NIAID TB Portals Program (TBPP) [6] initiated the creation of a novel data repository containing socioeconomic, geographic, clinical, laboratory, radiological, and pathogen genomic information from deidentified patient cases. This TBPP initiative brings disparate, local, clinical records of DR-TB cases from countries burdened with TB to the attention of the global research community in the form of an open-access online resource. Clinical and microbiological information is augmented by the results of M.tb pathogen full genome sequencing and both expert- and computer-derived descriptors of clinical images (CXR and CT).

TBPP follows the recommendations of the NIH Big Data to Knowledge (BD2K) initiative to enable biomedical research as a digital research enterprise [7]and adheres to the NIH FAIR principles [8]. The TB Portals Program is included in the US National Action Plan for Combatting MDR-TB [9]; the NIH Strategic Plan for Data Science [10]; and the NIAID Strategic Plan for TB Research [11]. By supporting a data ecosystem that accelerates discovery as part of the translational medicine, NIAID helps countries with a heavy burden of DR-TB to coordinate their efforts and share unique patient-centric data to shape future research with practical implementations.

As of November 2018, TBPP has collected 2,428 total cases from nine country sites (Azerbaijan, Belarus, Moldova, Georgia, Romania, China, India, Kazakhstan, and South Africa), 1,611 (66%) of which are multidrug- or extensively-drug resistant and 1,185 (49%), 863 (36%), and 952 (39%) of which contain X-ray, computed tomography (CT) scan, and genomic data, respectively.

TB Portals Data Browser (https://data.tbportals.niaid.nih.gov), consolidates all TB Portals Program data. On this site, users can search for defined clinical cases using multiple keywords or browse and review selected patient case histories. All patient cases are anonymized, entered, checked for quality issues, and released for publication with the help of a data capture module, which accumulates a growing list of clinical, socioeconomic, image, and genomic data. The TB Portals contain patient-centric data, which means that we do not augment our records with externally downloaded information; every piece of information in the record is related to a specific patient’s diagnostics, treatment, monitoring, and outcome. From the beginning, TBPP was selective, specifically choosing patient cases with the most complete information from multiple domains. There are currently 170 data descriptors, making the resource unique in enabling wide-ranging data mining and hypothesis testing.

TB research focused on imaging or genomic data analysis has recently achieved significant success [12, 13, 14, 15, 16, 17, 18, 19, 20, 21, 22, 23, 24]; however, there are still few investigations that encompass clinical, socioeconomic, image, and genomic data. Most TB databases and resources are domain specific, including ReSeqTB [25], PATRIC [26, 27], TB DB [28], ChestX-ray8: hospital-scale chest X-ray database [29] and others. Frequently, an existing resource or database may contain genomic information; however, socioeconomic information is missing, and the outcome of the treatment is unknown. This situation does not allow for detailed analysis of multiple factors that may influence epidemiological and clinical patterns of the disease. For personalized treatment—treatment that considers the pathogen’s resistance profile, the host’s metabolic profile, as well as the immune system’s readiness, the presence of other diseases or chronic conditions, and the state of lungs, etc.—a database is needed with sufficient information related to all of these factors.

For such unique requirements, we did not find an available solution to the challenge of TB data organization and analysis. It became imperative to develop an application that could traverse all data attributes to execute meta and cross-disciplinary analysis.

In this manuscript, we introduce the TB Data Exploration Portal) (TB DEPOT, https://depot.tbportals.niaid.nih.gov) to address the need to easily access clinical, radiological and genomic patient data and instantaneously execute multi-domain hypothesis creation and testing. We built TB DEPOT as a web- and cloud-based advanced analytics application that allows analysis of rich metadata from distinct domains of knowledge. It provides user-friendly, intuitive methods of combining, visualizing, and interpreting heterogeneous descriptive data. This approach, with the fulfillment of FAIR principles and aspects of the BD2K initiative, may be expanded toward other pathogens, data domains, and research settings. Here, we summarize the structure and programmatic usage of TB DEPOT, review the results using example case studies, and discuss DEPOT’s applicability for medical training and clinical and research purposes.

## Materials and methods

### Data preparations

Data for TB DEPOT is extracted from the TBPP database, which is described in Rosenthal *et al* [6]. The TBPP database is a de-identified, properly curated, and physician validated, published, trusted accessible resource. TB DEPOT aims to provide access to “analysis-ready” data and the ability to generate and test complex clinically-oriented hypotheses instantaneously with minimal statistical background and data processing skills.

TB DEPOT consolidates variations in temporal records related to a patient case history into a single data record. For data that are collected over-time, the value of a field is set using the ‘if ever’ principle: if a descriptor (like usage of a particular drug or drug sensitivity test outcome) is/was ever present during the course of a patient case, then it is recorded. For instance, if a single drug was used and then the drug regimen was changed, and the drug was no longer used, the drug is recorded as being present within the analysis dataset. Multiple drugs, markers, and other clinical and radiological descriptors may change during TB treatment; the ‘if ever’ principle ensures the information is not lost and may be utilized by TB DEPOT.

In TB DEPOT, a patient case corresponds to the data describing a single episode of active TB disease beginning with diagnosis and following through treatment. All resources contained within TBPP utilize a controlled vocabulary of terms commonly used and recommended by professional organizations and regulatory bodies including the WHO, CDC, NCBI, and others. The complete original patient cases are accessible within the TB Portals database and may be viewed with the TB Portals Data Browser (https://data.tbportals.niaid.nih.gov).

### Image annotation

Clinical lung images are a distinct category of data contained within the TB Portals database that TB DEPOT utilizes. At the time of this writing, there are 1,185 cases with chest X-rays (2D images) and 863 cases with CT scans across 2,428 patient cases. Like other types of information within the TB Portals, all images and their descriptors (either single or multiple) are associated with a specific patient case.

The TBPP utilizes expert annotations and computer-generated descriptors to convert images into data suitable for statistical comparison. Both an expert radiologist and machine learning methods may annotate frontal chest X-ray (CXR) and/or CT images using a standardized set of features like cavities, nodules, darkening of the lungs, etc. These image annotations are mostly equivalent to the information that radiologists routinely provide to practicing physicians. This process effectively converts the lung image into one or more categorical variables suitable for statistical analysis and comparison with other images.

### Image similarity

TB DEPOT utilizes image-based search functionality. This enables both descriptor- and image-based searches as they fulfill distinct needs. Image-based similarity searches are more general and not limited to a defined set of descriptors (mentioned above). One may start with general image similarity and then further restrict the search with a chosen set of descriptors. Also, external users have the ability to upload their own images, for which we may not have a compatible set of descriptors; hence image similarity is the only method to identify matching images from the complete set of patient cases.

General digital image features such as intensity, local binary patterns, gradients, and orientation were computed for all CXRs. The images with like distributions are considered “similar” [30]. There are many ways to approach the computation of similar images using specific feature emphasis related to a specific research question. For example, the overall size and shape of the lung could be important to estimate physical performance of a subject, while the presence and/or location of similar cavities and nodules may become critical for identifying similar pathology.

TB DEPOT enables searching for the most similar images, starting with any image in our collection, or by selecting a user-submitted image. In this way, TB DEPOT serves as a virtual assistant that can retrieve all of the details from thousands of patients’ case histories. While the TBPP currently does not offer recommendations or diagnostics for any user-submitted cases, the TB DEPOT does provide the gateway to detailed patient case records, which may inform practicing physicians or students regarding treatment plans, side effects, outcomes, etc.

### Creation of genomic SNP vector to characterize drug susceptibility

The TB Portals contain genomic data derived from whole-genome sequencing of 952 *M*.*tb* isolates from sputum and surgical samples. These sequences are scanned using standard bioinformatics algorithms to identify single nucleotide polymorphisms (SNPs) and other relevant genomic descriptors such as digital spoligotyping, lineage, and strain. We also mapped a subset of SNPs previously demonstrated as critical for drug resistance to all genomic samples. Specifically, we selected SNPs identified and described by the ReSeqTB database as either “large and often conclusive evidence” or “moderate evidence for drug resistance.” TB Portals continuously contributes genomic data to the ReSeqTB effort and will update these SNPs of interest as ReSeqTB publishes new analysis results [25, 31].

The decision to focus on an abbreviated, clinically-relevant SNP-based similarity rather than evolutionary relevance, was due to the clinical and logistical practicality of this approach. The genome of *M*.*tb*, is approximately 4.4 million nucleotides long, with over 4,000 genes (open reading frames) within it, and many of these genes have not been experimentally characterized and do not have well-characterized homologs from model organisms. This amount of information could be overwhelming and is unnecessary for a clinician who is primarily interested in knowing whether a particular pathogen will respond to a certain drug. Within TB DEPOT and G-AP we chose a binary vector representation approach to compute genomic similarity through clinically-relevant SNPs. Presenting the entire bacterial genome as a collection of SNPs relevant to antibiotic resistance serves two purposes. First, it effectively reduces the amount of information to its most relevant components, intuitively understandable to every clinician. Second, it enables the introduction of the “drug resistance similarity” profile based on commonalities in genomic factors responsible for drug resistance.

Reflecting the presence or absence drug resistance-associated SNPs, a binary vector is created for each annotated genomic sequence. If a specific SNP was present, a score of 1 is given for that SNP, and a 0 if it is absent. These single-digit scores are then concatenated into a “SNP vector” for each sequence. This vector approach distills a large volume of genomic data to those elements that are essential for understanding *M*.*tb* resistance to existing drugs. These vectors may be easily updated to include additional new SNPs as well as the presence or absence of other genomic markers. The Euclidian distance between binary vectors serves as a numerical score of drug resistance similarity between two genomes. This approach (creating numerical vectors thus greatly reducing the object size) might be uniformly applied to characterize presence or absence of any genomic markers and is flexible enough to accommodate new knowledge of SNPs responsible for drug resistance.

### Cohort creation

For the purposes of this paper and referring to current nomenclature used in TB DEPOT, we use the term “cohort” liberally, to refer to groups or subsets of patient cases selected in TB DEPOT by the user. Our usage of “cohort” here and in TB DEPOT does not refer to the epidemiological definition, in which observation time is defined and incorporated, but simply a group of patient cases with a defining characteristic(s).

TB DEPOT’s cohort creation functionality enables user-driven identification and review of specific or groups of cases. Scenarios that utilize this capability are diverse, including hypothesis creation and testing, clinical education, referencing, and consultation. TB DEPOT currently provides two principle means for identifying a cohort–Query Builder and Similarity.

**Fig 1:**
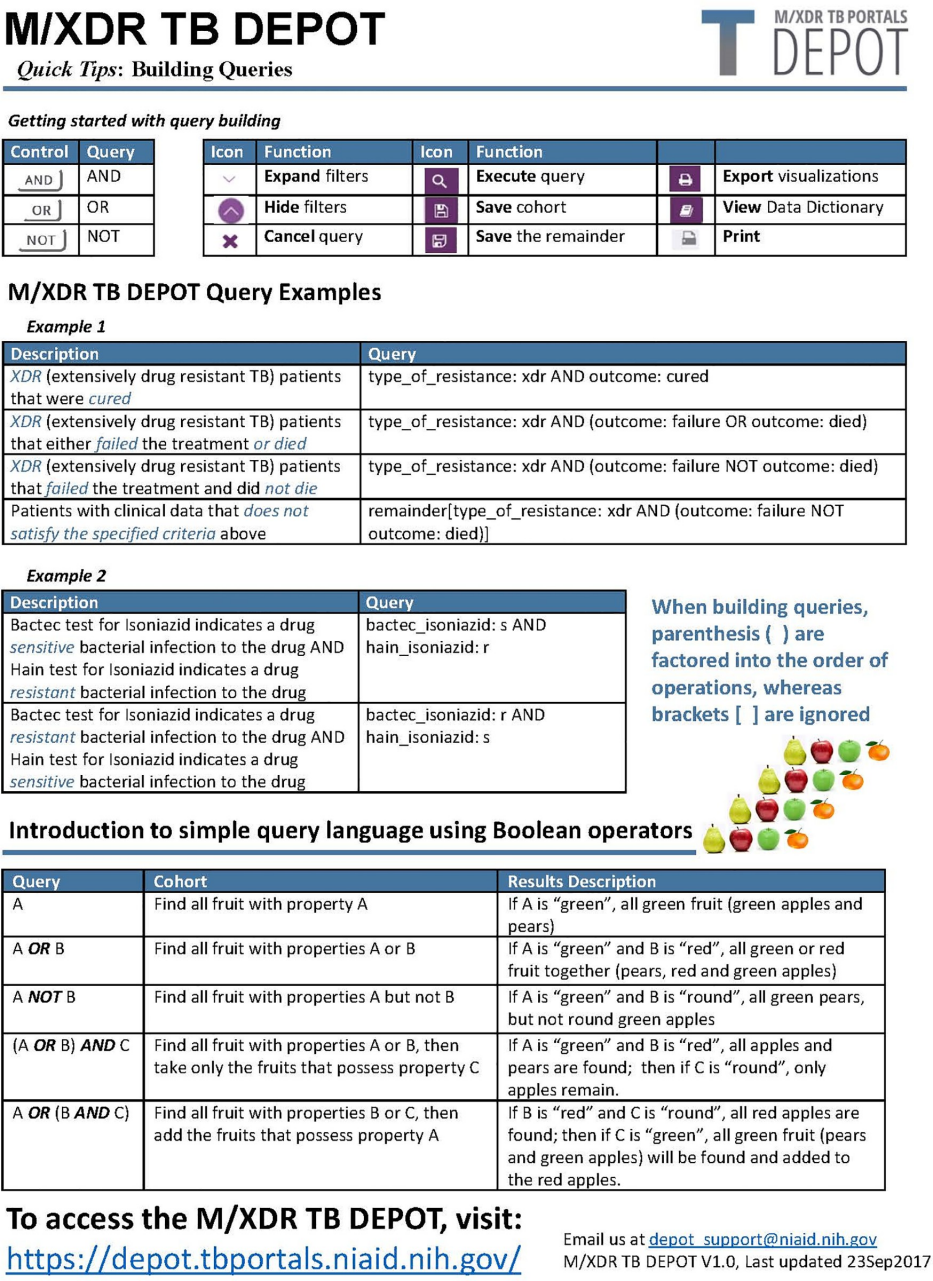
TB DEPOT tip sheet.

The Query Builder loosely follows the syntax of NCBI PubMed queries, where query terms can be combined using Boolean logical operators. Our reasoning behind this choice was that most medical professionals and scientists use NCBI PubMed and are already proficient with this type of querying [32]. Fig 1: TB DEPOT Tip Sheet provides some examples of TB DEPOT specific descriptors used with the Boolean operators ‘AND’ and ‘OR’ to create a query. When building queries, parentheses are factored into the order of operations, whereas brackets are ignored.

Similarity analysis provides an alternate means of creating cohorts through identification and selection of patients with similar lung image or pathogen’s genomic data. An important distinction between these two methods is that “similarity” initially provides an ordered list and the user must decide upon a threshold to define the size of the cohort.

To simplify working with TB DEPOT and sharing the most interesting results with colleagues, users can save queries and cohorts for future reference and repeated analysis. A saved cohort consists of the query statement and the associated patient case identifiers. Query language statements are preferable to button or checkbox selections because the query statements can be stored, re-executed, and easily shared.

### Visualization of key features during cohort creation

TB DEPOT implements a set of pre-defined graphs to visualize the most important clinical descriptors: resistance, outcome, case definition, comorbidity, gender, body mass index, and age of onset. At the start of a user session, all graphs are related to the publicly available TBPP dataset. As soon as the first query term is introduced and executed, the changes are reflected in horizontal bar charts showing the counts and percentages of the selected versus non-selected patient cases. This provides immediate visual feedback on the composition of the derived cohort and assists in keeping cohort counts large enough for statistically significant comparisons.

### Statistical analysis of cohorts

After the cohort(s) are formed, the user can view or save the corresponding data records or proceed to statistical analysis. TB DEPOT implements contrast analysis; i.e. finding asymmetries in the distribution of features between two cohorts.

TB DEPOT conducts analysis with both categorical and continuous data descriptors. Categorical data descriptors include, for example, outcome, drug resistance profile, and lineage. Continuous data descriptors include a range of numerical data, like body mass index, number of children, age of onset, and number of daily contacts. Contrast analysis of categorical attributes is performed using Fisher’s Exact Test, while continuous attributes are assessed using Wilcoxon rank-sum test. The unadjusted p-values from these tests are aggregated, sorted, labeled and summarized using the common thresholds indicating a level of statistical significance.

TB DEPOT has integrated PLINK V1.9, an open source genome association analysis toolset to identify the most common genomic variants between genomes from distinct cohorts (groups) [33,34]. PLINK considers all SNPs in the entire M.tb genome found by the variant calling pipeline, thus executing genome-wide association (GWAS), enabling the identification of novel variations that may explain the development of TB drug resistance and other clinically relevant features.

### Visualization of results for cohort analysis

Cohort analysis results can be explored through several views in TB DEPOT. Under Summary View, results are presented in a ranked order by statistical significance. Categorical and Continuous views allow users to compare cohorts of interest using the pull-down lists of categorical and continuous variables, in which distributions and frequencies are shown for each variable. Tree diagrams and box plots are used to display these results under this view. Under the Scatterplot tab, a user can plot a categorical variable against a continuous variable. Kaplan-Meier plots display a comparison of time to outcome of interest curves between cohorts. Start of treatment is normalized for patients in each cohort, with treatment start day set as time zero.

Genomic View displays the list of genes containing the most significantly different SNPs between the two cohorts. SNPs are plotted against their genomic positions as a graph reflecting the significance of differences between cohorts. In addition, the distribution of SNPs within cohorts can be viewed in a table ordered by statistical significance and accompanied by relevant genomic information (gene name, genomic position, cohort sample size, and type of mutation).

### System implementation

TB DEPOT was constructed with a flexible architecture and extensible back-end database design. We implemented TB DEPOT using custom Microsoft.NET code for the web interface, PostgreSQL for the database, Highcharts v6.1.1 for data visualization with Javascript charts, and R v3.2.5 for statistical computation and analysis. Both Microsoft Windows Server 2012 R2 and Linux Ubuntu are leveraged to host the application on six servers within the Amazon Web Services cloud. TB DEPOT is a publicly-accessible web site that does not require authentication; however, for saving, reusing personal queries, and running comparative analyses researchers are able to register with their e-mail address. Additional algorithms contributed by our collaborators are incorporated at separate Portals (Genomics, Radiomics) and those used by DEPOT are connected using secure web services. A more detailed description of the technical approach used to build TB DEPOT is the subject of a separate paper (in preparation).

## Results

### Multidisciplinary data access and analysis

**Fig 2:**
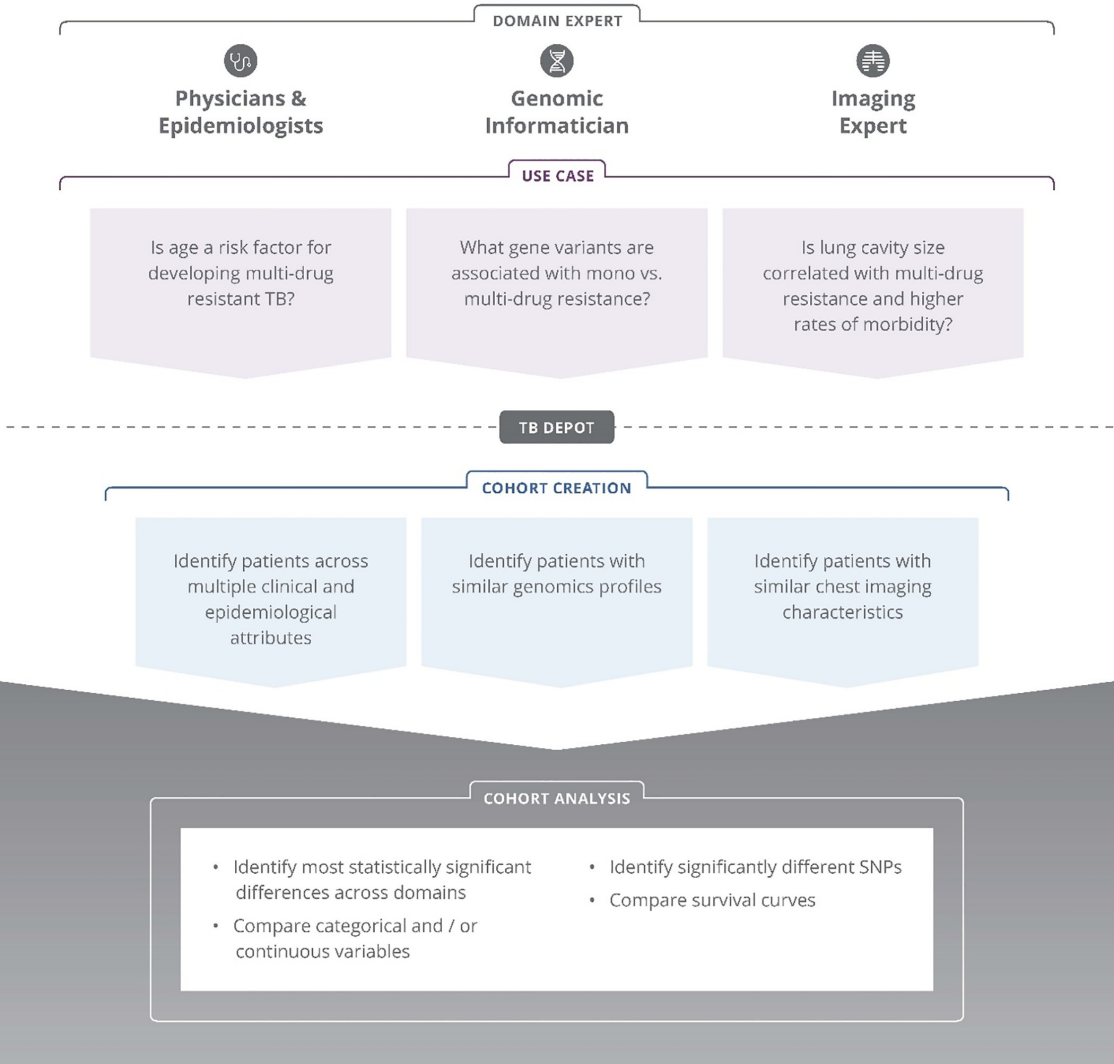
TB DEPOT example use cases.

TB DEPOT provides physicians, epidemiologists, genomic informaticians, and imaging experts with a web-accessible resource to instantaneously generate and test hypotheses across a rich collection of socioeconomic, clinical, imaging, and genomic TB patient case data. Several use case examples are shown in Fig 2. These use cases illustrate the line of logic used to develop inquiries into hypotheses that can be readily tested via cohort creation and analysis. TB DEPOT can be used for many purposes, including:

- searching for individual cases and groups of patients possessing one or more indexed characteristics;
- forming patient cohorts based on patient case histories–as if consulting with a referenced patient case folder;
- visualization of the distribution of features within the cohort by choosing the appropriate tab for clinical features, lung images, or genomic data;
- comparison and statistical analysis of any two cohorts selected from the collection of patient cases; and
- visualization of the comparison results.

It should be noted that results obtained from the analysis of TB Portals data may or may not be further extrapolated to the entire TB population; the data collection was primarily aimed at MDR-TB and XDR-TB patient cases. Consequently, percentage-wise, TBPP collection is not representative for epidemiological studies without proper adjustments.

Results of hypothesis testing are generally not intended for direct use in the diagnosis or treatment of a disease or medical condition. Using a link labeled ‘disclaimer’ appearing in the footer on each page of the web application users are advised to consult with a qualified physician for diagnosis and for answers to personal health questions.

### Engaging with TB DEPOT

**Fig 3:**
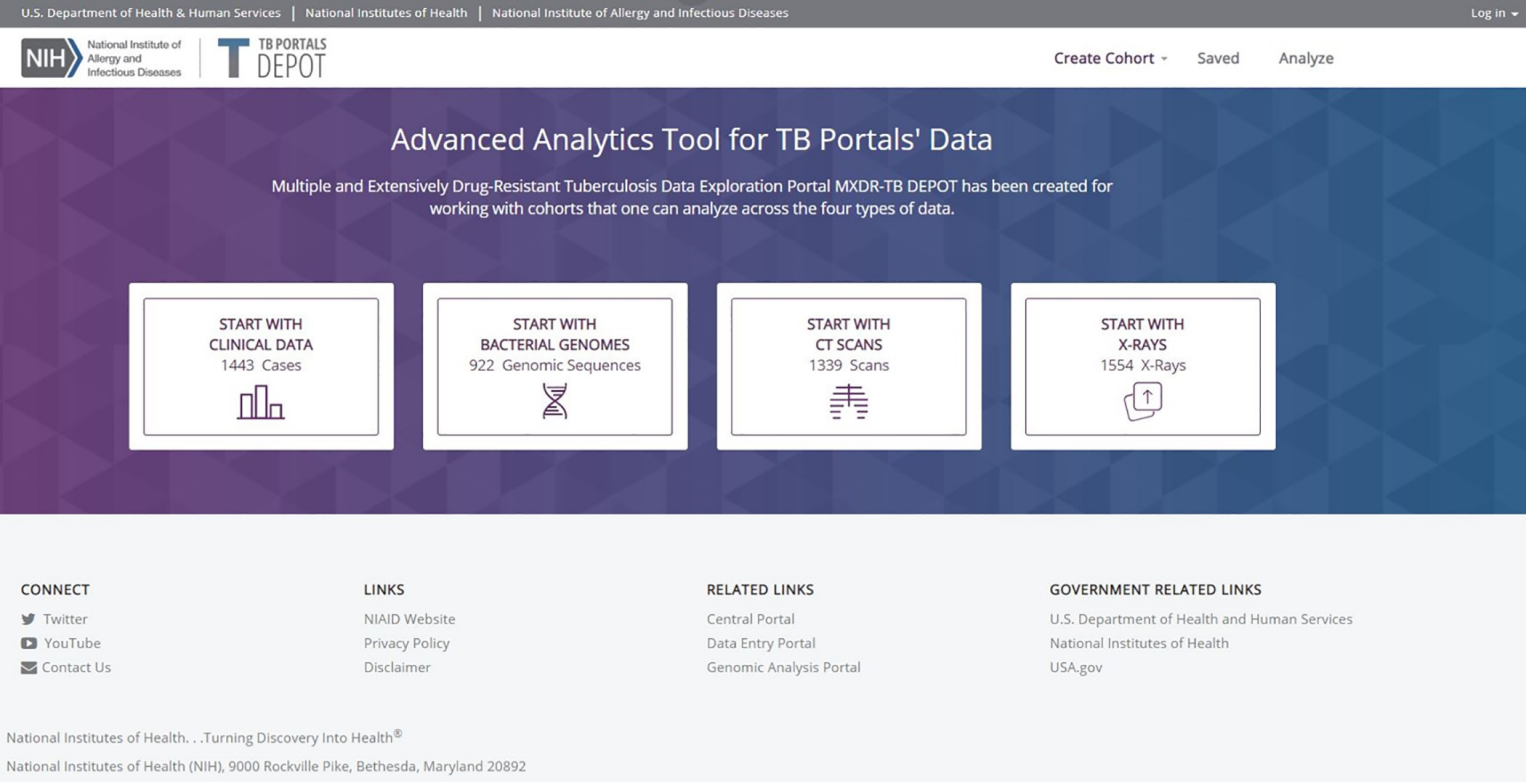
TB DEPOT landing page.

The TB DEPOT landing page (https://depot.tbportals.niaid.nih.gov) provides starting points for engagement with the tool (Fig 3). The interface is designed so that users can quickly choose the specific class of data they prefer to work with: 1) *clinical data*; 2) *bacterial genomes*; 3) *CT scans*; or 4) *X-rays*.

Here, we present three case studies to demonstrate aspects of TB DEPOT functionality and explore key features in more detail. We will demonstrate the following steps in TB DEPOT usage: 1) formation of cohorts; 2) visualization of data; 3) saving cohort data; 4) statistical analysis of two cohorts; and 5) presentation of results.

#### Case Study #1: Seeking radiological features that may be related to treatment outcomes in XDR-TB patients

In this first usage scenario, TB DEPOT helps to analyze radiological annotations correlating with treatment outcomes. TB DEPOT can be used to find XDR-TB patients with accompanying image and outcome data, as there could be multiple factors contributing to or associated with a negative outcome. Identifying the most important and manageable parameters may help physicians to choose the most effective treatment plans.

For this analysis, all patients with XDR-TB that also had accompanying clinical image data will be identified and then divided into two groups: XDR-TB patients that were successfully treated, i.e. cured, and XDR-TB patients that failed treatment or died. To create these two cohorts and execute a comparative analysis, the user will enter the TB DEPOT application using the “*Start with Clinical Data*” selection from the login screen (Fig 3). The user is then invited to create a cohort using the query builder. Under the query builder search text box, the data domains are shown as horizontal tab fields. The most common clinical attributes are shown in the left-hand column with the Boolean operators.

**Fig 4:**
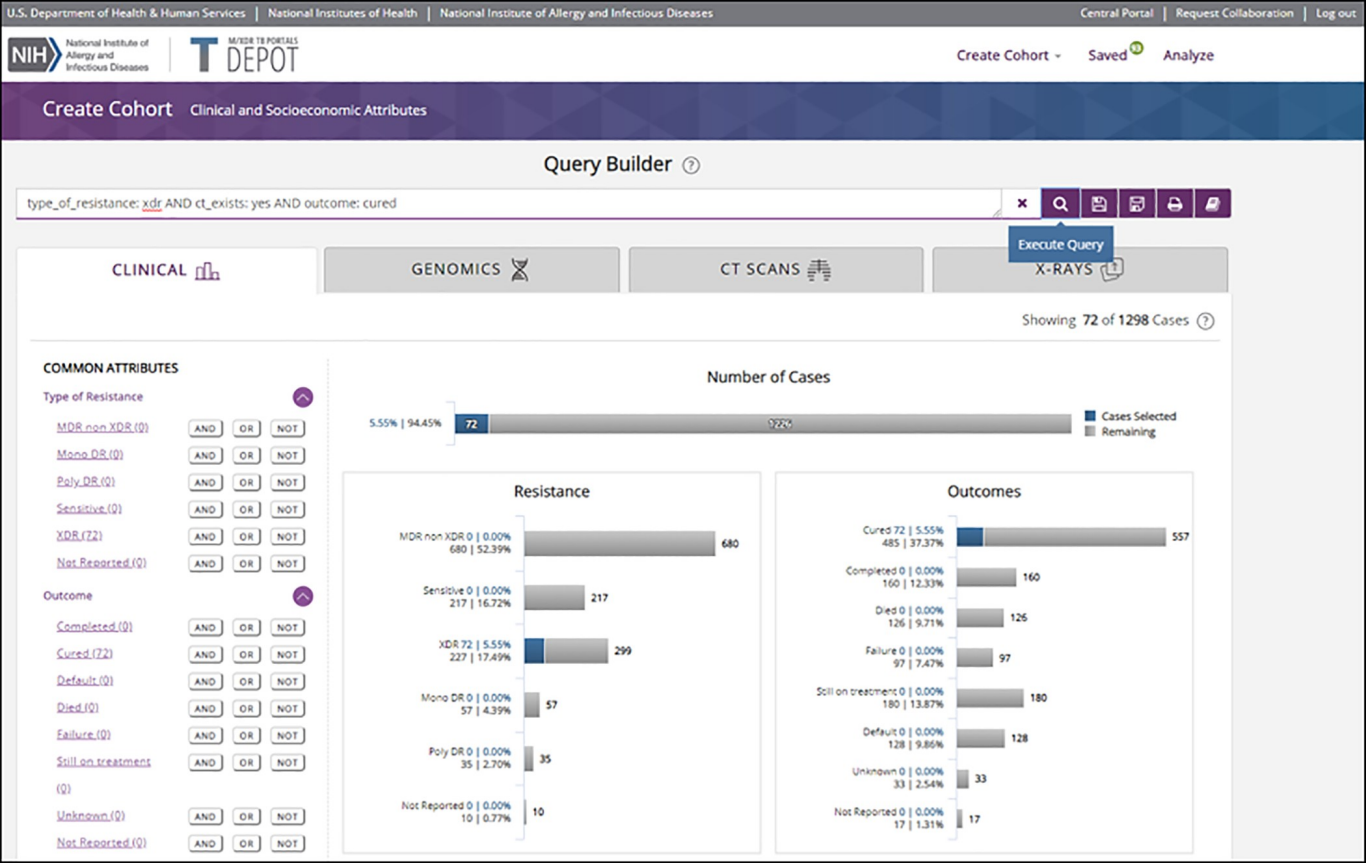
TB DEPOT Query for patients with extremely drug resistant tuberculosis, where CTs were available, and the reported outcome is “Cured”.

To select cases for the first cohort in this scenario, the user selects the “*Type of Resistance*” field drop-down and then selects “*XDR*” (Extensive Drug Resistance). If cases including CT scans are desired, the user would then move down to “*CT Image Exists*” and select the “*AND*” button adjacent to the “*Yes*” value field. Finally, the user selects the “*Outcome*” field drop-down and selects the “*AND*” button adjacent to the “*cured*” value field. The query was thus formed as shown in the screen capture Fig 4 TB DEPOT Query. During the creation of a cohort, type of resistance and outcome are displayed for user visualization and guidance. The query is then executed by using the “*Execute Query*” magnifying glass icon shown to the right of the query search text box. Cases that match this query are displayed.

The number of cases that are extensively drug-resistant, have a CT image, and are cured is 72, and there are 1,226 cases that do not meet these criteria. The horizontal bar charts for Resistance and Outcomes show these data with additional context and detail (Fig 4).

**Fig 5:**
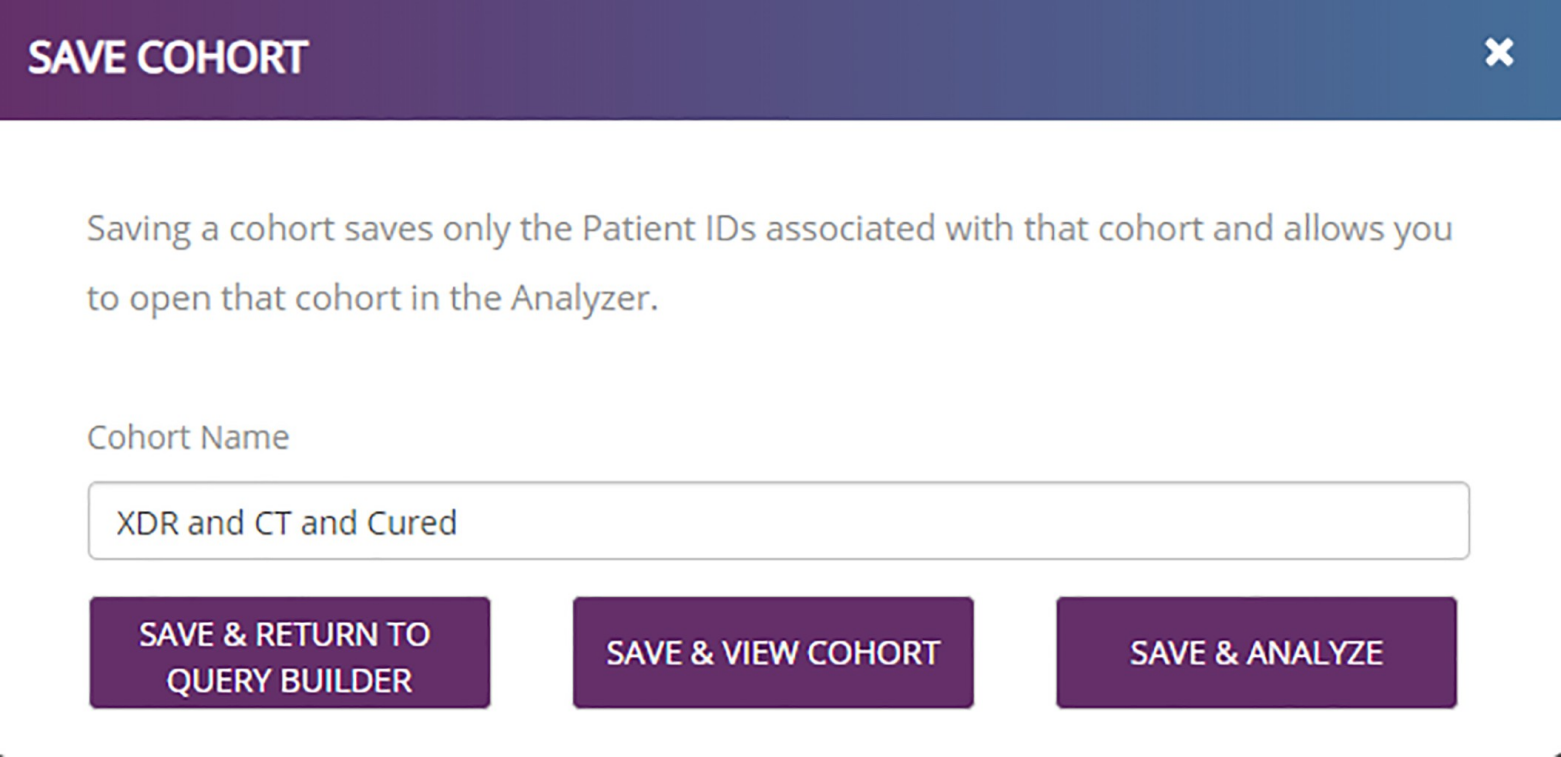
TB DEPOT Save the cohort consisting of patient cases with extremely drug resistant tuberculosis, where CTs were available, and the reported outcome is “Cured”.

The user may then save the cohort after providing an appropriate name. An example is shown in Fig 5. In this session, the user selects the option to save the cohort and return to query builder. Within the query builder, the comparison cohort—those XDR-TB patients with a CT image that failed treatment or died—is then created using terms “type_of_resistance: xdr AND ct_exists: yes AND (outcome: failure OR outcome: died).” With this second cohort created, the user may then select the “*SAVE & ANALYZE*” option shown in the lower right of Fig 5.

**Fig 6:**
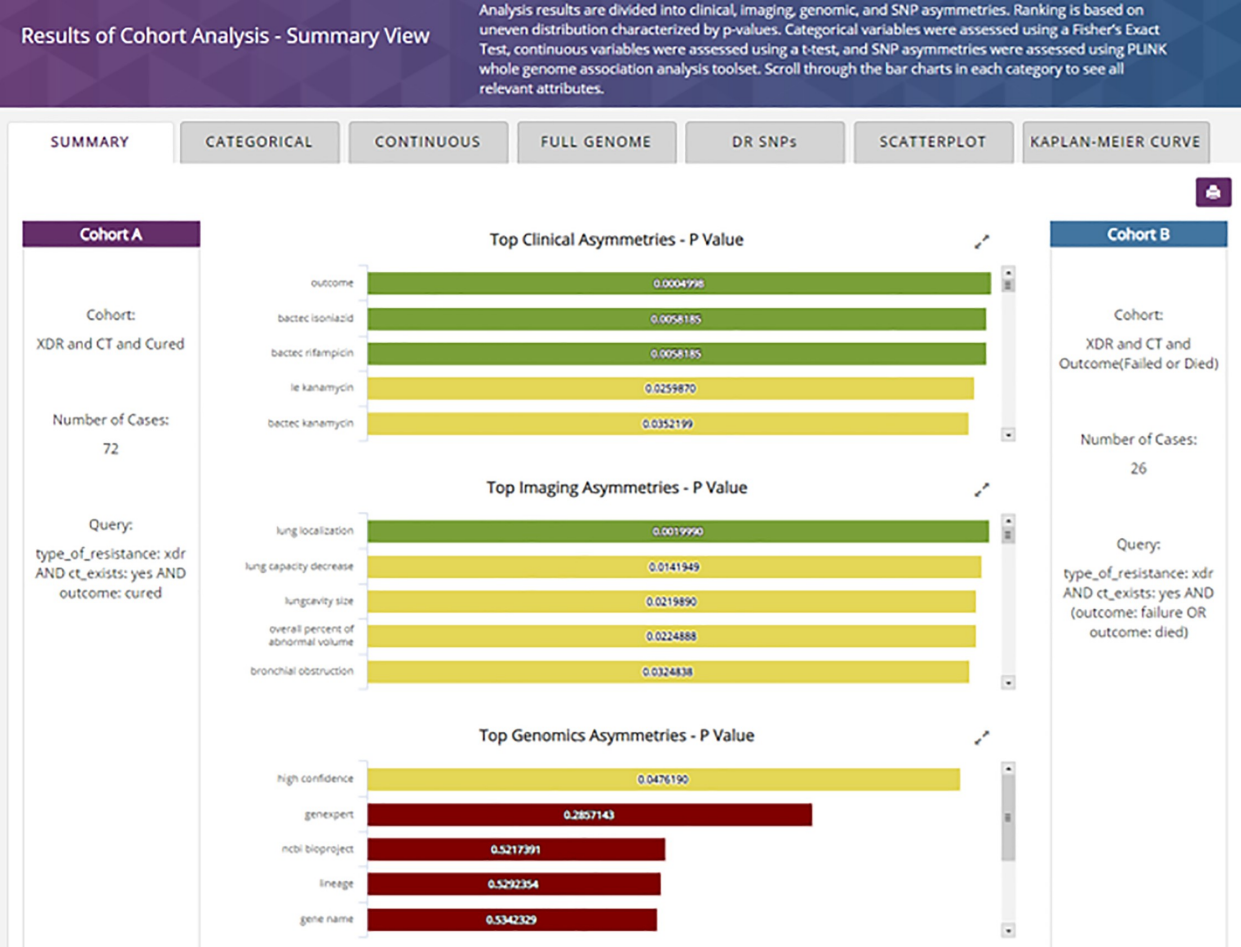
TB DEPOT Cohort Analysis—Summary view.

By selecting the “*SAVE & ANALYZE*”, or by selecting the “*Analyze*” menu button available in TB DEPOT menu, the user chooses the two cohorts for analysis and initiates processing (not shown). The results within the cohort analysis summary view are then displayed. The summary results are shown in four vertical sections, only three of which appear in Fig 6 Summary View.

After the execution of the analysis, the Summary View is presented with horizontal tabs covering the breadth of currently implemented comparisons and analysis available within the TB DEPOT. Within the Summary tab specifically, results from each data field under Clinical, Imaging, Genomic, and SNP asymmetries are shown in order of statistical significance, with their respective P-value. The color of each bar is graduated from green to red to show the decreasing order of significance. For the comparison of the above-mentioned cohorts, within the Top Clinical Asymmetries section, the statistically significant differences are “*outcome*”, “*bactec isoniazid*” and “*bactec rifampicin*”. Among the Top Imaging Asymmetries, the most significant P-value is for “*lung capacity decrease*” (p = 0.0142).

**Fig 7:**
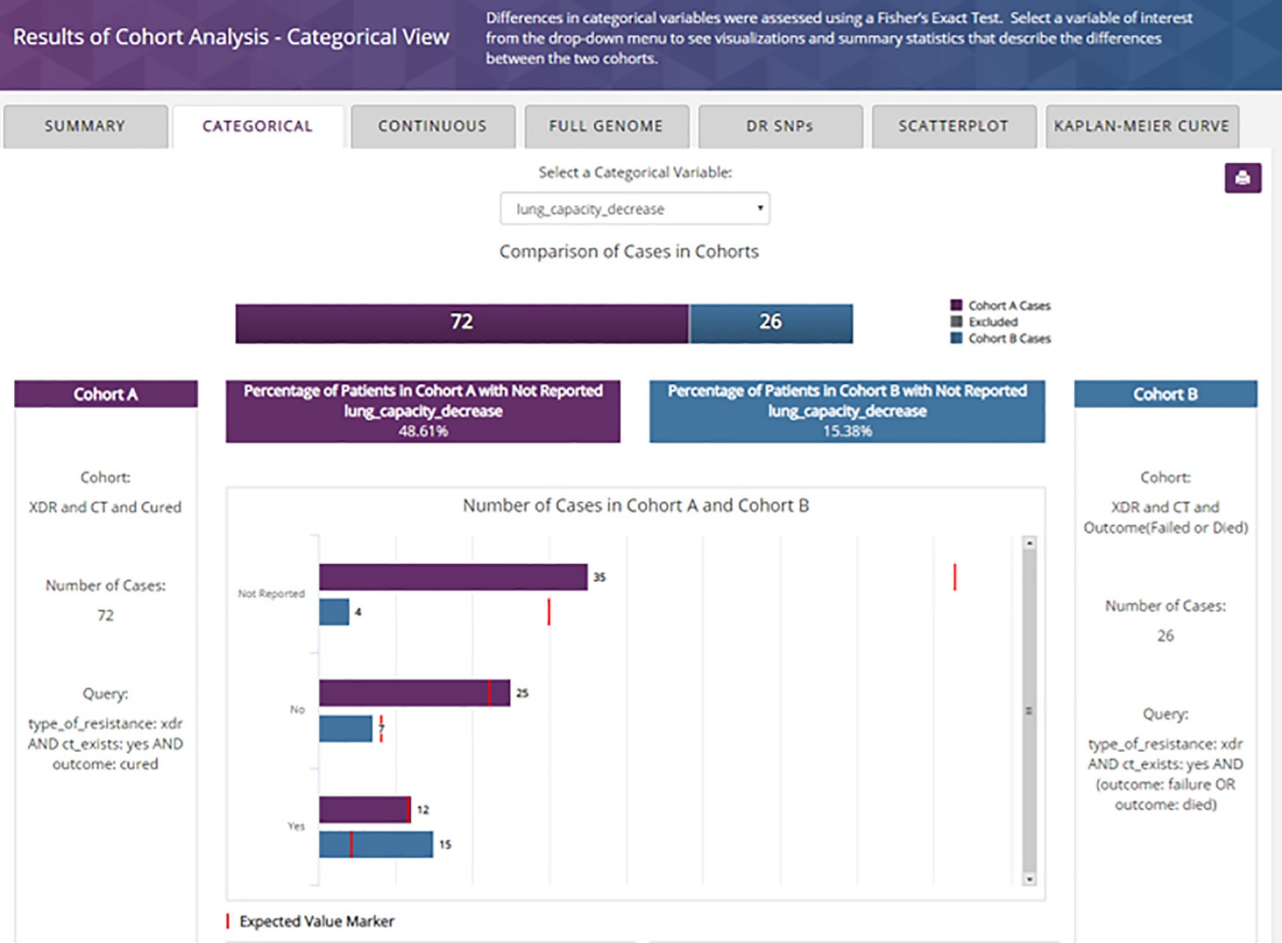
TB DEPOT cohort analysis–Categorical view, showing the comparison of “lung capacity decrease”.

Using the *Categorical View* tab, the user selects “*lung_capacity_decrease*” from the drop-down and generates the following details shown in Fig 7. XDR-TB patients that have died or failed treatment and for whom CT data exists are more likely to have lung capacity decrease than those that were cured and had CT data.

Using the *Continuous View* tab, the user may select fields from the drop-down menu and generate graphical displays with appropriate visualizations and a table of detailed statistics. None of the continuous fields were statistically significantly different between these two cohorts.

TB DEPOT analytics illustrate the differences between Cohort A: XDR, CT exists and Cured (72 cases) and Cohort B: XDR, CT exists and Failed or Died (26 cases). While the results of this analysis are specific to the TB Portals dataset this finding does agree with results from Chen et al. describing the predictive capacity of CT scans and the sensitivity of some radiological markers in distinguishing successful versus unsuccessful TB treatment [13].

#### Case Study #2: Using image similarity analysis to identify and review patient cases

TB DEPOT can be used to find patient cases with similar lung images and enable advanced visualization and analysis of associated clinical records. The similarity comparison can be initiated with a user-submitted image or from an image contained within TB DEPOT’s indexed collection of images. In this second example, we demonstrate how TB DEPOT’s similarity function enables the user to search for similar frontal lung X-ray images (CXR), to review the detailed case histories, and to create cohorts comprised of patient cases with these similar images.

To upload an X-ray image, the user will navigate from the landing page (Fig 3) by selecting “START WITH X-RAYS”. The “Create Cohort” screen (Fig 4) will appear with the “X-RAYS” tab selected (not shown). The user selects the “UPLOAD X-RAY IMAGE” button to browse their local file system and select the file containing the frontal CXR. The image file is then uploaded and the search for similar CXRs is executed.

The image comparison is executed based on an algorithm developed by Kovalev et al. [35] and adapted by Juarez-Espinosa et al. [30]. The set of patient cases related to the returned similar images may be treated as a cohort or may provide the starting point for subsequent similarity searches.

**Fig 8:**
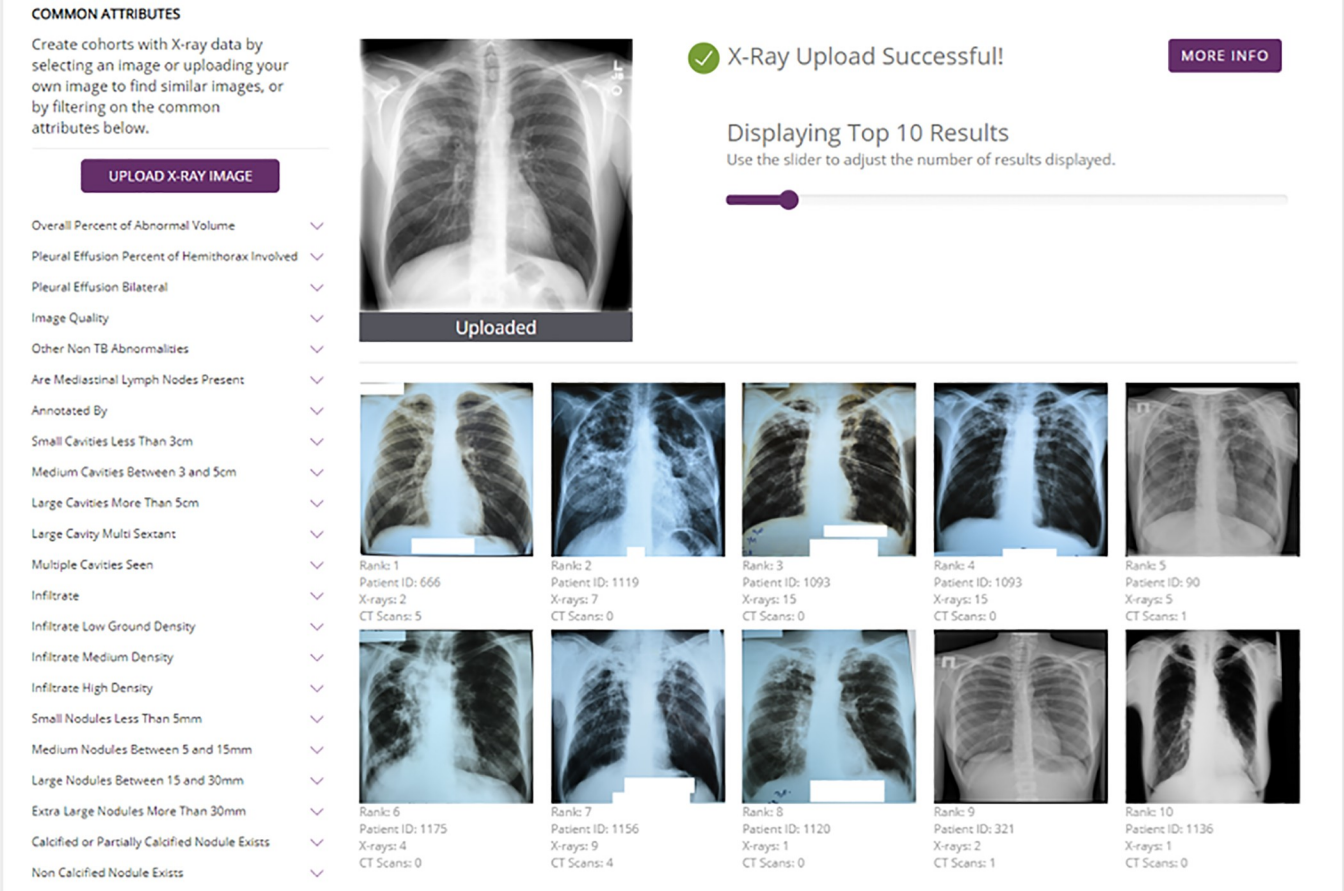
TB DEPOT Using the uploaded X-ray image to perform a search for similar images in the database.

In Fig 8, the similarity function was applied to a random uploaded image (found using Google image search with the keyword “cavity mass”). The user can browse the images returned by TB DEPOT’s similarity analysis function and read accompanying patients’ clinical histories, as if he/she was consulting a referenced patient case folder. In this example, TB DEPOT identified similar CXRs from nine annotated patient cases. Among these patient cases, there are two XDR-TB cases, six MDR-TB cases, and only one drug-sensitive patient found to have similar CXR images.

Importantly, all associated patient data from cases in this cohort created by the image similarity function are available to the user for review by clicking on the tabs for Clinical features, CT Scans, or Genomic features. Finally, the user may save and use the cohort of the original X-ray image and most similar X-ray images to conduct comparative analysis against other cohorts. This approach is not only useful for clinical reference purposes, but it also aids hypothesis creation that could be followed-up in future clinical research studies.

#### Case Study #3: Comparing bacterial genomic sequencing results for patients with drug resistant and sensitive tuberculosis

TB DEPOT has two distinct methods for comparing genomic features between cohorts. First, a classic genome-wide association study (GWAS) algorithm can be utilized in TB DEPOT [33, 34]. The advantage of GWAS analysis is that we can study any problem dependent on genomic variability. For example, we might compare SNP differences in geographically distinct regions or study correlations with macroscopic events like culture growth.

Second, as will be shown in this case study, we apply a body of knowledge that currently exists for SNPs conferring specific known resistance to anti-tuberculosis drugs (DR-SNPs) [25]. It has been reported that widely used microbiological drug-sensitivity testing may be inconsistent [36]. The use of full genome sequencing with subsequent mapping of drug resistant SNPs could help in resolving contradictions between microbiological testing methods. The “computed” drug resistance profiles for sequenced pathogens will become commonplace as the sequencing of bacterial genomes continues to diminish in price and the technology proliferates. The genome-based approach is not a complete substitute for microbiological methods because resistance to specific second- and third-line drugs is still not fully explained based on genomic information alone [37].

To verify the presence of documented known DR-SNPs within TB DEPOT data, we compared two cohorts identified by type of resistance. The first cohort was formed from patient cases that are sensitive to tuberculosis drugs. The second group consists of cases with drug-resistant tuberculosis (DR-TB) of any type as defined by the WHO [38]: mono-resistant, poly-resistant, multidrug-resistant, or extensively drug-resistant.

**Fig 9:**
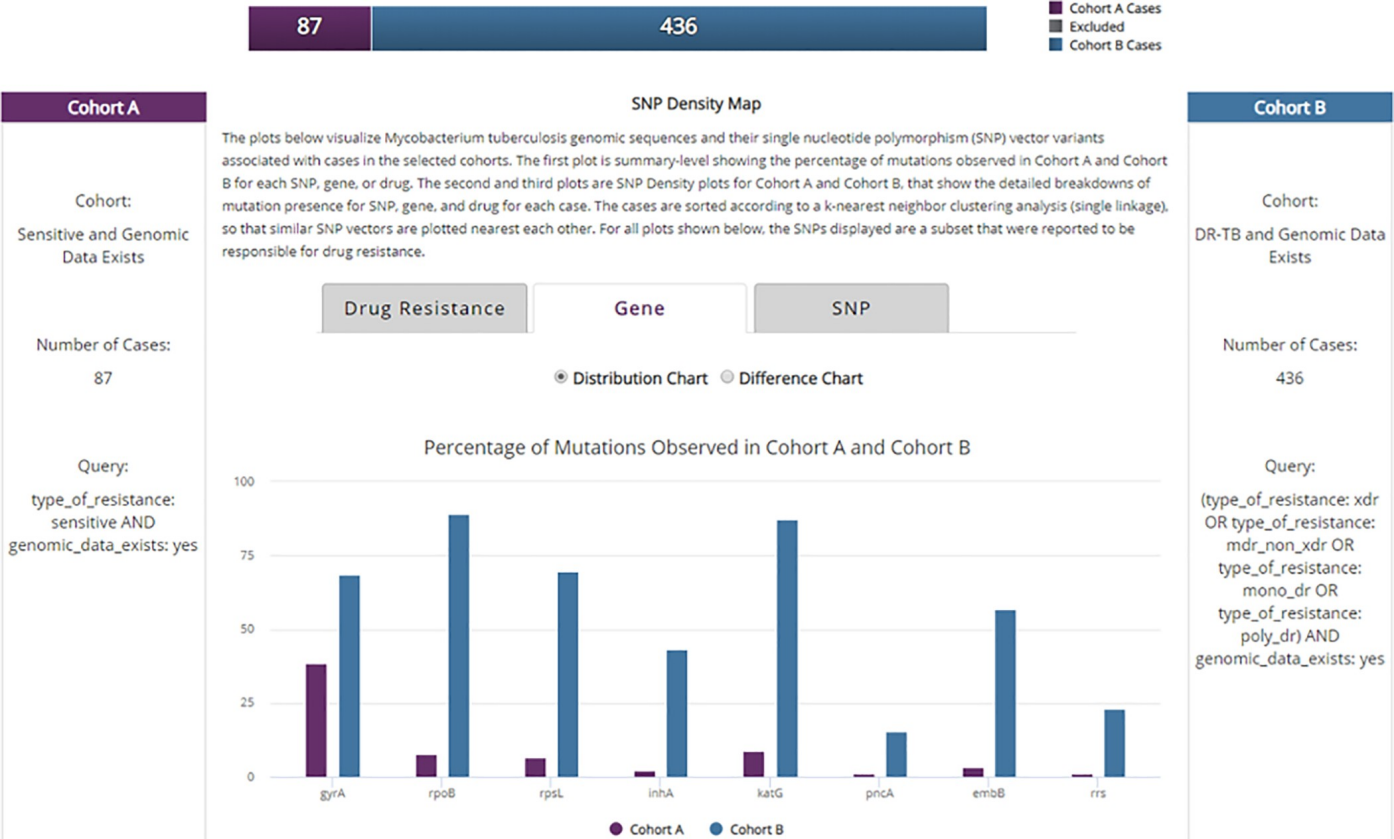
TB DEPOT Analysis summary results: genomic mutations in Sensitive vs Drug Resistant Tuberculosis (DR-TB) cases.

As shown in Fig 9, Cohort A has 87 patient cases with drug sensitive TB, and Cohort B has 436 patient cases with DR-TB disease; both cohorts only include cases that contain associated genomic data. Fig 9 shows the histogram of relative frequency of occurrence for the most prominent DR-SNPs [25].

As expected, most known DR-SNPs were found in genomes among the DR-TB patient cohort, with 5 of these SNPs appearing in more than 50% of DR-TB patient cases. The specific relationship of gene to drug resistance is summarized in Table 1. Except for gyrA, the presence of these SNPs in the genomes of drug-sensitive patient cases is negligible and could be cases of mixed infections.

**Table 1:** Gene/SNP associated with resistance.

Gene Affected	SNP incurs resistance to*
gyrA	Isoniazid
rpoB	Rifampicin
rpsL	Streptomycin
inhA	Isoniazid
katG	Isoniazid
pncA	Pyrazinamide
embB	Ethambutol
rrs	Amikacin, kanamycin, capreomycin

*As described by the ReSeqTB database (https://platform.reseqtb.org) as having either “large and often conclusive evidence” or “moderate evidence for drug resistance. ”

We further studied the distribution of drug-resistant SNPs for separate groups of patients chosen by country by DR profiles, age groups, and outcome of the treatment (manuscript in preparation).

## Discussion

The TB Portals Program’s introduction of the Data Exploration Portal successfully fulfills the need for a multi-domain hypothesis testing and analysis engine. It furthers the TBPP goal to provide a complete web-enabled ecosystem of resources, critical for countries with a high burden of TB but with limited IT resources. TB DEPOT effectively leverages the TBPP integration of clinical, socioeconomic, imaging, and genomic data. It expands the ability to search and visualize aggregate user-selected groups of patient cases. Experts from different areas can explore, create, search for, and share defined cohorts from a large and growing body of data collected by TBPP.

### Benefits

With TB DEPOT, it is possible to leverage the functionality and data in many ways, for many distinct clinical, teaching, and research settings. Here, we will discuss the many benefits of TB DEPOT that are widely applicable and can be further configured in accordance to expert-specific needs.

TB DEPOT can be used when a practicing doctor or researcher must quickly examine statistics from a specific hospital setting, age group, or geographic area, providing the ability to easily separate the cases of interest from the entire patient case catalogue. Most hospitals and scientific institutions have their own separate databases storing clinical, socioeconomic, and other patient data. The identification and analysis of patient groups requires someone with database processing skills. In addition, execution of statistical analysis requires additional knowledge and experience to process source data into formats suitable for statistical applications. TB DEPOT provides access to “analysis-ready” data and the ability to generate and test hypotheses instantaneously with minimal statistical background and without the prerequisite of data processing skills.

#### Bridging multi-domain knowledge areas

Doctors and researchers may begin the analysis of various groups of patients starting with features that correspond to their own background or field of interest. They may, for instance, want to examine *M*.*tb* strains with a certain type of drug resistance or high virulence. After full genome sequencing is done and bioinformatics data processing steps identify genomic variability and make-up, these data become available for TB DEPOT’s integrated PLINK tool to perform genome wide association studies (GWAS). The purpose of GWAS and the mapping of known drug resistant SNPs onto collected genomes enables the identification of those SNPs that appear within one cohort but not another. Because genomic data, like all data within TBPP, is linked to patient case clinical information, subsequently one can review the inferences from genomics to the most important clinical parameters.

TB DEPOT can also be used to challenge, confirm, and augment subject matter expertise. This is especially important as knowledge of sensitive TB can be fine-tuned for the emerging threat of MDR and XDR tuberculosis. The results of comparison over all metadata fields may not only support or reject the initial hypothesis, but, hopefully, prompt a researcher to engage in additional ‘omics testing, radiologist to review the clinical images, or a physician to think about modifying a treatment strategy.

**Medical training resource.** TB DEPOT can greatly enhance medical training by making many well-annotated, hard-to-find patient cases accessible. With the help of TB DEPOT, TBPP content may be utilized as a growing detailed electronic physician-curated resource, as it provides complete patient case studies including hundreds of images, volumes of genomic data, and treatment outcomes. Conventional printed materials are necessarily limited in amount, recency, granularity, and the diversity of examples. Through TB DEPOT and TBPP, medical instructors can highlight a multitude of patient cases, diagnostic approaches, treatment plans, prognosis, and outcomes.

**Simplified Advanced Analytics.** The inappropriate use of antibiotics contributed to a health crisis related to drug-resistant pathogens, subsequently precipitating calls for action [39, 40, 41, 42, 43]. Optimization of treatment for drug-resistant pathogens and mixed infections may require full genome sequencing, knowledge of specific markers from the host genome, as well as the monitoring of the host’s microbiome. TB DEPOT is specifically geared toward expert-driven investigations that compare drug regimens, diagnostic tools, radiological information, and genomic variations of pathogen between cohorts of successfully and unsuccessfully treated patients. To this end, TB DEPOT exhibits the following features:

- a modern, intuitive, and powerful approach to enter, store, index, search, and visualize patient cases;
- the ability to create, save, and share groups of patients with common characteristics (virtual cohorts);
- analysis of cohorts and user-selected groups, using the appropriate statistical methods;
- investigation and display of trends in underlying data;
- providing immediate testing of hypothesis;
- investigating treatment success rates through survival curve analysis; and
- furnishing print-ready graphic visualizations and statistics.

#### Uniformity and completeness of analysis

One of the core ideas behind TB DEPOT was that the goal for optimizing diagnosis and treatment begins with a tool that ranks all collected parameters (normally used by doctors) in the context of their relationship with treatment outcome and/or clinical manifestation of the disease. Across a wide range of parameters, TB DEPOT uniformly applies the same set of statistical criteria to present a sorted list of all parameters, starting from those most asymmetrically distributed between chosen cohorts (the “contrast analysis”). In this way, TB DEPOT works without preconceptions about the importance of one factor over another for specific cohorts of patients or types of pathogens.

As such, TB DEPOT is a helpful tool for researchers trying to examine hypotheses of clinical importance or relevance over a wide range of parameters. Its ability to perform meta-analyses may justify further research and analysis, potentially outside of the initial query domain or user’s field of expertise. As with all hypotheses testing, correlations do not imply causations, and many DEPOT results may initiate more thorough controlled clinical studies with additional patients’ recruitment and other types of experimental data collected.

### Future objectives

#### Growing database and extending TB DEPOT

Modern technologies are influencing the diagnosis and treatment of TB patients. The body of knowledge regarding the genetic make-up of patients, genomic information of the pathogen, and metabolism of drugs by applying ‘omics approaches is growing. TBPP and TB DEPOT are well positioned to absorb and effectively analyze ever-increasing types and volume of data.

TB DEPOT stores both static query builder definitions and the associated patient case identifiers as an excellent means to document the analysis steps, share the virtual cohort definitions and aid collaborative work. For example, the logical query text may be published in a manuscript, shared in email, or included in a PowerPoint presentation as readable and re-usable text. The data associated with the virtual cohort may be exported. As the size of the database grows, the same query text may be re-executed to bring additional new patient case records. In future releases, we plan to permit users to recognize any amended or updated patient case information.

TB DEPOT is not a bioinformatics analysis tool, nor is it designed to investigate radiological images. Computations of genomic variability, automatic annotation of images, and any domain-specific analysis is delegated to other components of the TBPP ecosystem. TB DEPOT’s specific purpose is to allow the user to form groups of patients and then perform a contrast analysis of distributions of clinical, radiological, genomic or other parameters in the chosen cohorts. The user is responsible for cohort formation, because the computer-generated exhaustive list of all possible cohorts would be unusable due to its’ size and lack of clinical relevance.

#### Genomic similarity

The Genomics Analysis Portal (G-AP, https://gap.tbportals.niaid.nih.gov) supports many features related specifically to genomic analysis. A manuscript describing G-AP as a part of the TBPP is being prepared. G-AP currently incorporates three open source algorithms for predicting drug resistance based on genomic information. One distinctive feature of G-AP that will be leveraged within the TB DEPOT is the ability to use genomic information to perform similarity analyses. A future version of TB DEPOT will include a user configurable genomic similarity function that is flexible to reflect specific requirements for the user’s inquiry into drug resistance, taxonomy, or other genomic markers.

#### Image similarity beyond human detection

TB DEPOT and the TBPP will increasingly employ published computational methods for image annotation and clinical feature predictions. As the complete set of all images are fully described with a uniform set of digital features, it becomes possible to compare images and identify a subset of features related to the progression of the disease, effectiveness of the treatment, etc. as demonstrated by, for example, by Chen et al [13].

This electronic analysis approach is complementary to human annotation, as it can identify features and patterns not detectable by the human eye. Also, it does so agnostically, without bias of what features are important from the human radiologist’s point of view. As the image resolution increases beyond the ability of the human eye to perceive, the possibilities for computer-aided diagnostics and prognostic tools will also increase.

#### Extending similarity as a general search method

Since the TBPP collects and stores a significant amount of well-organized, uniformly-annotated, and freely available patient-centered information, it can be used as a reference for doctors treating new TB patients. Relying on clinical histories from TBPP assumes that people with similar histories (socioeconomic), clinical (pre-existing conditions), genomic (similar metabolic and immune system markers), and pathogens (same TB strains, similar DST profiles) might react to treatment in a similar manner. Lungs with similar damage might require reconsideration of treatment options. Pathogens with closely related genomes may interact with our immune systems in a similar way. A physician’s goal would be to choose the most efficient strategy of treatment based on collected information about the patient and the pathogen. If he/she can find similar cases with positive outcomes, then those cases can be used as a reference.

Because of its ability to identify and retrieve similar data based on known and potential clinical relevance, TB DEPOT also excels as a tool to aid clinicians in treating their patients. Standard clinical cases are widely and routinely used during medical education to illustrate certain important points for the treatment of patients. It is, however, impossible for physicians to keep in mind hundreds of patients, their clinical data, images, and genomic make-up for later reference. Instead, TB DEPOT can quickly analyze this breadth of information and properly identify similar cases from these types of data.

#### TB DEPOT time-series analysis

Currently, TB DEPOT is oriented to a snapshot, rather than continuous time-bound patient case data. Future releases will incorporate multiple time points in treatment including changes in drug regiments, tracking lung feature changes, and multiple genomic samples.

## Conclusions

As demonstrated, TB DEPOT, as part of the TB Portals Program, further fosters collaborative research and clinical efforts to better understand DR-TB and aids in the development of novel diagnostics and personalized treatment regimens. The utility and practical applications of TB DEPOT’s querying and analysis functions are diverse, supporting a range of clinical and research applications for a variety of end users. While developed specifically for the TB Portals’ growing multi-domain TB dataset, it could also be applied for use in other medical research fields. In turn, TB DEPOT can continue to expand through exchange with other bio- and clinical informatics disciplines, incorporating additional functionality and ultimately resulting in a more innovative, interconnected ecosystem with novel applications for research and precision medicine.

Addressing the growing threat of MDR-TB requires a comprehensive understanding of the disease, which could be achieved by multi-center, global collaborations. The TB Portals Program was established with this mission in mind, consolidating curated and de-identified patient socioeconomic, clinical, radiological, and genomic information from TB cases. TB DEPOT was developed as a unique tool to utilize the wealth of metadata contained within the TB Portals, readily displaying and analyzing it to facilitate hypothesis generation and testing, aimed at improving MDR-TB patient diagnostics and outcomes. It is a powerful, user-friendly, and open-access tool that can be used by a wide variety of end users, but especially TB researchers and clinicians. It can help physicians develop more targeted TB treatment regimens and improve existing ones; it can reveal interesting research questions for researchers to pursue. TB DEPOT remains a tool in active development, constantly evaluating and adopting: new types of data, new algorithms for analysis, and new data exchange protocols, to better answer the needs of physicians, researchers, and domain specific experts.
